# Co‐producing an inclusive‐care model for young people transitioning from adolescent eating disorder services to adult care: A qualitative study protocol for Transition for Eating Disorder Youth intervention

**DOI:** 10.1002/erv.3046

**Published:** 2023-11-07

**Authors:** Maria Livanou, Anya Heneghan, Elli Bouliou, Grace Hill, Katharine Mills, Sophie Naylor Roll, Zara Smalley, Jun Teh, Janet Treasure

**Affiliations:** ^1^ Department of Psychology School of Mental Health & Psychological Sciences Institute of Psychiatry, Psychology and Neuroscience King's College London London UK; ^2^ Department of Psychology Institute of Psychiatry, Psychology and Neuroscience King's College London London UK; ^3^ Department of Psychological Medicine Institute of Psychiatry, Psychology and Neuroscience King's College London London UK

**Keywords:** anorexia nervosa, bulimia nervosa, childhood, risk factors, treatment

## Abstract

Eating disorders (EDs) present a chronic issue to child paediatric mental health services due to their high mortality and relapse rates. The transition from Child and Adolescent Mental Health Services to Adult Mental Health Services is a particularly high‐risk period for young people with EDs given their high vulnerability to change, which can negatively impact treatment outcomes. However, there is lack of evidence on the feasibility of inclusive and youth‐specific interventions that address the multiple and complex needs of this group during their transition to adult care. This proposed study aims to develop a newly introduced model of care called Transition for Eating Disorder Youth intervention (TEDYi) for young people aged 16–18 years with EDs transitioning from adolescent ED services to adult care. TEDYi will be co‐produced with young people, carers, and staff targeting interpersonal and psychosocial needs during the transition process. The first phase of the study involves interviews with young people (*N* = 15) and carers (*N* = 15) as well as focus groups with mental health professionals (*N* = 15) across four ED adolescent and adult specialist services to explore their transition experiences. The second phase, consists of four Experience‐Based Co‐Design workshops, aimed at collaboratively developing and refining TEDYi.

## Highlights

- The transition from Child and Adolescent Mental Health Services (CAMHS) to adult care represents a challenging period for young people with eating disorders (EDs) due to their vulnerability to change, which has been identified as a significant predictor of relapse and poor treatment outcomes.
- Young people with EDs as they navigate emerging adulthood are likely to experience additional transitions pertinent to residential, occupational, and developmental changes, identity reconstruction, change in therapeutic models from CAMHS to Adult Eating Disorder Services (AEDS), which often elevate the risk of experiencing social isolation.
- Optimal transitional care with developmentally appropriate interventions which emphasise emotional readiness has the potential to improve prognosis, increase engagement and provide ongoing support.
- Experience evidence‐based interventions informed by personalised guidance can empower young people and their families, and those involved in service delivery by facilitating understanding of their roles and increasing self‐management of symptoms.
- There is very little evidence about the feasibility and effectiveness of inclusive and youth‐specific interventions that address the complex needs of this group as they transition to the community to prevent adverse mental health outcomes.
- To develop meaningful evidence‐based interventions, and to improve transitional processes and outcomes for young people moving from adolescent care to adult services, it is pivotal to involve young people with lived experience in the design and production of research projects.
- This protocol outlines the process we followed to develop a series of empowering workshops to create the *Transitions for Eating Disorder Youth intervention* (TEDYi) to support young people aged 16–18 years with anorexia (AN) and/or bulimia nervosa (BN), and other comorbid mental health problems, and their families, as they transition to adult care.

## 1 INTRODUCTION

Eating disorders present a significant public health concern and pose a chronic issue to child paediatric mental health services due to their high mortality and relapse prevalence (Walker & Lloyd, 2011). Eating disorders are life‐threatening diseases which can result in premature death among young adults, with the highest mortality rate among all psychiatric disorders and they are linked to poor psychosocial outcomes, mental health comorbidities and repeated hospital admissions (Berends et al., 2018; Cardi et al., 2017; Treasure et al., 2011). An estimated one‐third of all deaths in young people with anorexia are due to cardiovascular issues, and 25% of this population have attempted suicide. Anorexia (AN) and bulimia nervosa (BN) are the most prevalent types of EDs among young people, and they exhibit alarming symptoms ranging from starvation to purging and using laxatives to excessive exercising. Eating disorders typically first appear in adolescence and early adulthood (Arcelus et al., 2008) where two thirds of cases continue for 9 years and a third for over 20 years (Eddy et al., 2017).

Young people often present to Child and Adolescent Mental Health Services (CAMHS) with overlapping symptomatology of AN and BN due to the diagnosis complexity underlying EDs. A study found that the mean age for young people presenting in CAMHS is 14.6 years and only 27.7% are transitioned to adult services (Arcelus et al., 2008). However, a considerable proportion is not accepted by adult specialist services and might be seen by generic services or treated in primary care, which is worrying due to lack of staff training and skills around EDs (Winston et al., 2023). Studies show that young people with EDs are more likely to experience poor transitions due to lack of continuity of quality care and fragmented social support in the community (Bryan et al., 2022). Young people experiencing poorly managed transitions which do not promote graded and flexible pathways are less likely to take ownership of their condition and, therefore become more prone to poor mental health outcomes (NHS, 2019). A large‐scale national survey identified substantial barriers to ED transitions, including the absence of personalised transition plans and limited involvement of carers. Additionally, the survey highlighted a therapeutic disconnect between child and adult care plans, as adult services often lacked a developmental approach to treatment (Winston et al., 2023).

The Royal College of Psychiatrists (2022) and NHS England (2015) advocate for prioritising ED transitions and improving discharge support, aiming to reduce relapse, optimise self‐efficacy, and enhance quality of life for young people. The transition from CAMHS to adult services is a particularly high‐risk period for young people with EDs due to their vulnerability to change, which has been identified as a significant risk factor for relapse and poor treatment outcomes. In fact, anorexia was initially conceptualised as a response to change stemming from overwhelming emotions (Bruch, 2001). Young people with EDs during emerging adulthood are likely to experience additional transitions pertinent to residential, occupational, and developmental changes, identity reconstruction, change in therapeutic models from CAMHS to AEDS, which often increase the risk for social isolation (Webb & Schmidt, 2021). Due to the high mortality rates and associated physical health complications, well‐planned transitions in individuals with EDs are pressing. Optimal transitional care with developmentally appropriate interventions has the potential to improve prognosis, increase engagement and provide ongoing support (Wales et al., 2021). Experience evidence‐based interventions can empower young people as well as their families, and those involved in service delivery by facilitating understanding of their roles and increasing self‐management of symptoms. Previous research highlights that guided self‐help interventions lead to poor outcomes underlined by low participation and high dropout rates and, therefore personalised guidance can enhance motivation and engagement (Giel et al., 2021). The debilitating effects of anorexia and BN are linked to family dependency and poor psychosocial functioning in the community.

The commissioning framework for ED services in England acknowledges the importance of including individuals with EDs in the development and implementation of care approaches and interventions. However, initiatives for young people specifically with EDs co‐producing care approaches and interventions are currently lacking (Lewis & Foye, 2021). Co‐production is a form of patient and public involvement (PPI) that is widely used in health research (Hayes, et al., 2012), where service users, family members, carers, and service providers collaborate to improve their own care and service provision (Norton, 2019). Experts by experience can identify needs, care gaps, meaningful outcomes, ultimately increasing transparency and improving care (Madden et al., 2020). A recent report published by the Beat Eating Disorders charity (2020) voicing the lived experiences of people with EDs and their carers, echoes the infrastructural barriers to transitions and the lack of a standardised transition plan with 50% describing the process as either “extremely poor” or “poor”. The lack of effective communication coupled with abrupt transitions prompted recommendations for further family involvement to prevent exacerbation of ED behaviours.

There is very little evidence about the feasibility and effectiveness of inclusive and youth‐specific interventions that address the complex needs of this group as they transition to the community to avoid preventable death causes. Therefore, this study aims to develop a series of empowering workshops to create the *Transitions for Eating Disorder Youth intervention* (TEDYi) to support young people aged 16–18 years with AN and/or BN, and other comorbid mental health problems, and their families as they transition to adult care. This approach aims to reduce anxiety and increase self‐efficacy (symptom self‐management) by building on existing skills and promoting hope. Evidence‐based research in this area is considerably lacking and we need interventions targeting transition preparations of young people moving to adult services at least 6 months before discharge (Arcelus et al., 2008). TEDYi will be person‐centred and co‐produced with young people (who have moved to adult services or are approaching 18 years and are due to transition from adolescent ED specialist services), carers and staff (nurses, nutritionists, family therapists, psychologists, psychiatrists, occupational therapists, GPs) with lived ED transition experience targeting interpersonal and psychosocial needs during the transition process.

The intervention will be developed collaboratively with young people, carers, and mental health professionals, integrating their contributions during the co‐production phase. We have outlined a preliminary intervention plan guided by the literature and previous evidence‐based supplementary interventions designed for adults. However, we aim to receive feedback on this proposed framework and make necessary adjustments and modifications, based on the perspectives, and lived experiences of the involved stakeholders. This process ensures that TEDYi is aligned with the needs of the target population. We aim to determine the frequency, mode of delivery and training materials included in the intervention based on previously conducted Experience‐Based Co‐Design (EBCD) workshops. Experience‐Based Co‐Design enables service users, families, and staff to work collaboratively, using the participants' lived experience, to co‐produce service design and contribute to research development. Experience‐Based Co‐Design has been implemented successfully in breast and lung cancer patients across two London teaching hospitals (Tsianakas et al., 2010) and in diabetic ED patients (Zaremba et al., 2022). We aim to co‐produce training materials for TEDYi including a handbook guiding the workshops and an accompanying animated video showcasing interactions of young people with peers and carers who have experienced transition. Young people will prepare for transition 3–6 months before discharge between services. This preparation aims to prevent relapse and manage the coexistence of physical and mental health symptoms.

## 2 THEORETICAL FRAMEWORK

Transition for Eating Disorder Youth intervention will be informed by previously effective intervention transition models‐ECHOMANTRA and TRIANGLE‐ for adult patients with EDs. ECHOMANTRA is a skills‐training intervention for patients and carers aimed to sustain recovery after discharge based on the interpersonal cognitive model (Treasure & Schmidt, 2013) which proposes that improving patient and carer communication will lead to better transition outcomes (Adamson et al., 2019). ECHOMANTRA includes coping strategies, for recovered ED patients, towards reconstructing an identity that is not emanated from the ED. Previous research has found that individuals with AN are particularly vulnerable to family members' expressed emotions (overprotection and criticism), due to excessive worry and anxiety (Rienecke, 2018). Research shows that add‐on interventions such as ECHOMANTRA facilitates psychosocial functioning and improves outcomes for people with EDs and their carers (Cardi et al., 2017). Thus, the aim is to equip carers with skills that will foster positive emotional responses in young people with EDs. Furthermore, it is well‐established in the extant literature that carers are often excluded from adult care‐plans (Eisler et al., 2022), both due to confidentiality and expectations that individuals are well‐skilled to manage an independent lifestyle once they have transitioned. However, young people previously hospitalised in highly supportive settings transferred to adult care and/or community settings which follow a more independent care model, often lack life‐skills, and encounter major challenges in leading an adult‐like lifestyle (Webb & Schmidt, 2021). Research evidence suggests that carer involvement in young people's care journey has the potential to reduce loneliness, and social isolation by empowering the young person (Cardi et al., 2017). Thus, carers will be included in the co‐production plan to reflect on their lived experiences of transition and voice their needs. Mental health professionals will also be included to facilitate the transition process, specifically, the *letting go* period for the young person. Young people often build strong attachments to keyworkers in services and ending therapeutic relationships may be a triggering factor for relapsing during transitions.

Similarly, TEDYi will be developed in line with TRIANGLE's and ECHOMANTRA’S underlying aims of upskilling young people, carers, and mental health professionals during transition periods. TEDYi aims to use a similar format around graduate leave planning although in‐person workshops will be aimed to be delivered by a peer‐mentor. Drawing upon discussions and reflective dialogs with mental health practitioners, clinical academics, and individuals with lived experiences of EDs, alongside prior evidence‐based interventions, we have outlined the foundational principles guiding TEDYi. However, our objective is to elicit specific feedback on the proposed structure and delivery approach through the present co‐production and development phase involving young people, carers, and mental health professionals from adolescent and AEDS. Carers and adults with EDs valued ECHOMANTRA in terms of psychosocial skills gained and maintaining a recovery identity and they emphasised the need for more consistent follow‐ups post‐discharge. Therefore, TEDYi aims to include a peer‐support mentor with lived experience to run preparation transition workshops. The proposed intervention will focus on building hope, deconstructing a non‐ED identity, managing emotional responses to change, and encouraging service engagement and follow‐ups, restructuring mealtime and planning, involving a primary carer. In the long run, TEDYi seeks to comprehensively address the interpersonal, psychosocial, and emotional needs of both young people and carers throughout the transition process.

## 3 AIMS AND OBJECTIVES

In the present study we aim to:

- conduct interviews with young people and carers who will or have moved from adolescent ED units to adult services and the community within the last 5 years;
- conduct focus groups with mental health professionals (staff) with lived service transition experience, to explore their views and roles in the transition process;
- receive feedback on foundational TEDYi outline and implement suggested adjustments.
- co‐design TEDYi and develop, together with young people, carers, and mental health professionals (staff), the format, structure and materials included in the intervention based on EBCD workshops;
- explore recommendations for transitional care from young people, carers, and mental health professionals.

## 4 RESEARCH QUESTIONS

- What are the views and perspectives of young people, carers, and mental health professionals about transitioning from adolescent ED services to adult care?
- What are the personal reflections of young people, carers, and mental health professionals on the proposed content and structure of TEDYi?
- What changes do young people, carers and mental health professionals recommend to TEDYi structure, format, and delivery?
- How can TEDYi be improved based on personal lived experiences of young people, carers, and mental health professionals?
- How can carers be involved in transition preparation and planning?
- What factors should TEDYi entail for successful and sustainable implementation in adolescent services long‐term?
- What strategies can be employed to improve joint working between adolescent services and adult services and facilitate the promotion of TEDYi?
- What strategies can we implement to maintain engagement, participation and adherence in young people, carers, and mental health professionals?
- What measures can be put in place to provide optimal support to both carers and mental health professionals during the transition process?

## 5 METHODS

### 5.1 Study design

This study will use qualitative methodology to explore the complexity of transitions from adolescent to adult ED services. Qualitative methodology will provide a deeper understanding of young people's and carers' lived experiences which would not be encapsulated in quantitative research. The present study includes two phases: (a) interviews and focus groups, and (b) EBCD workshops. Young people who wish to be part of the wider co‐production group will be invited to the four follow‐up EBCD workshops. Focus groups will be used with mental health professionals as they are more familiar with sharing sensitive content in groups due to their profession (Patel et al., 2016). Interviews will be conducted with young people and carers to allow individual expression and avoid feelings of fear that may arise during sharing lived experiences in larger groups. The interviews will be recorded for use in EBCD workshops. Interviews and the four EBCD workshops will help the research team understand the priorities of young people and carers during the transition process and to address specific themes of concern.

### 5.2 1^st^ phase interviews and focus groups

- Four postgraduate research assistants, a Doctorate Clinical Psychology trainee and a doctoral researcher with lived experience will conduct the interviews and focus groups. All students have received qualitative training and have relevant clinical experience. All interviews will take place in the service the young people attend or online via Microsoft Teams depending on availability and personal preference. The interviews will include young people (*N* = 15) and carers (*N* = 15). All interviews will last 30–60 min. For the focus groups, we aim to recruit mental health professionals (*N* = 15) from adolescent and adult services and primary care (GPs). They will be separated into two focus groups both including mental health professionals across adolescent and adult services. All interviews and focus groups will be conducted by one moderator and a research assistant to observe, take field notes, and record any non‐verbal cues. The focus groups will last 60–90 min. Participants will receive a payment of £10 Amazon Vouchers for their participation in interviews. The participant information leaflet of the study and the researcher clearly state that if the participants choose to be audio or video‐recorded, we may use some of their quotes or video‐recorded material to create a short video that will be discussed during the subsequent workshop phase. To ensure confidentiality and protect participant anonymity, blurring faces will be used and no participant will be identified through the shared recorded materials. In case, participants do not wish to share any specific or sensitive information in the follow‐up phase, they will let the researcher know. However, recording interviews on both audio and video and potentially showing parts of these recordings in subsequent workshops could introduce concerns about participant bias and willingness to disclose sensitive information. To mitigate this risk, the research team will aim to create a trusting, inclusive and safe environment during the interview phase to encourage open and honest sharing of lived experiences. Researchers are well‐trained in reflexive qualitative approaches and have their own lived experiences which will empower the interview process. After the recording participants will have the opportunity to provide feedback or ask questions about the process to address any concerns they might have and reinforce the confidentiality measures. An independent reviewer of the team will contribute actively to the selection of recorded segments to be shown in the workshops.

The following predetermined codes will be used as prompts in interviews and focus groups: experience with services, current or past support throughout the transition process, barriers and facilitators in the transition process, continuity of care, effective communication, the role of staff in the process, recommendations for mechanisms in place, next steps. In the focus groups with the mental health professionals, we will also focus on the following: approaches to teamwork and decision‐making within and across services, resources to facilitate transition and continuity, role development, skills and training necessary to improve staff confidence in the provision of effective transition and continuity to users and carers. These codes were developed as a continuous process of collaboration between the team members, after a literature search and input from PPI members.

### 5.3 2^nd^ phase: Experience‐based co‐design workshops

Participants who wish to participate in the follow‐up workshops, will register their interest through the consent form. We will aim to recruit a representative sample across adolescent and adult services by including young people and carers who did not yet undergo the transition process as well as young people and carers who have already moved to adult services. Similarly, mental health professionals from adolescent and adult services may share conflicting experiences about transition processes, discharge preparation, and parallel care. The first group may primarily express uncertainties and fears, related to the transition process, while the second group may report on their specific (positive and negative) experiences during transition.

- Young people (*n* = 4), carers (*n* = 4), mental health professionals (*n* = 4) and members of the research team (*n* = 3) as well as the Principal Investigator (PI) will participate in the four EBCD workshops. Our aim is to involve the same group of young people, carers, and mental health professionals in all four EBCD workshops to maximise consistency and transparency. However, every participant will have the option to withdraw or choose not to attend subsequent workshops if they wish to do so.
- The PI and supervisor of the team will attend all EBCD workshops.
- All workshops will last 3 h with two 15‐min breaks and one 30‐min lunch break in‐between. The EBCD methodological approach includes 20 min of filmed content that highlights, from the interviews and focus groups, current service user transition experiences. Using PPI feedback, we will review the content and duration of the video and extract quotes that capture the transition process according to the lived experience of young people and carers. Content deemed as too sensitive will be excluded. Participants will be reimbursed with £30 for each EBCD workshop. These rates are based on PPI rates according to the National Institute for Health nad Care Research (NIHR) guidelines to acknowledge lived experience contributions in the project.

#### 5.3.1 1^st^ workshop: Discuss and reflect on the video content

- In the first part of the workshop (1.5 h), the participants will discuss the findings from the interviews and focus groups and how these can inform the intervention. During the second part of the workshop, the participants will watch the 20‐min video, taking notes throughout. The main themes presented will then be discussed and participants will be encouraged to brainstorm themes which they found important based on their lived experiences. The prominent ideas will be shared on a Padlet and used for the second workshop.

#### 5.3.2 2^nd^ workshop: Developing module themes for Transition for Eating Disorder Youth intervention

- The participants will reflect on the themes elicited from the 1^st^ workshop and then themes will be further developed on the premise of capturing the priorities and lived experience of transitioning from the perspective of the young people and the carers. The participants will collaboratively create an experience map outlining barriers and facilitators for an optimal transition experience. The initial design of TEDYi will be shown to the participants as it was first proposed by the research and clinical team. We will discuss the format and structure of the design and ways of increasing engagement.

#### 5.3.3 3^rd^ workshop: Adapting materials

- Participants will review the ECHOMANTRA documents and provide input regarding its adaptation in TEDYi. Psychoeducation materials are provided for both young people and their carers, who are also involved in their meal practice. The outcome measures of this intervention will be discussed in relation to transition preparedness, mental health symptoms (depression, anxiety, self‐harm, suicidality), physical symptoms (cardiovascular problems, electrolyte imbalances, hypoglycemia) and quality of life.

#### 5.3.4 4^th^ workshop: Meta‐workshop feedback

- The first part of this workshop will focus on reviewing and refining TEDYi content based on previous feedback. We aim to receive feedback on these specific outcome scales: Hospital Anxiety and Depression Scale (HADS; Zigmond & Snaith, 1983), Depression Anxiety and Stress Scales (DASS‐21; Lovibond & Lovibond, 1995), ED Symptom Impact Scale (EDSIS; Sepulveda et al., 2008), The Caregiver Skills Scale (CASK; Hibbs et al., 2015), Transition Readiness Assessment Questionnaire (TRAQ; Wood et al., 2014). These scales were utilised in previous studies exploring transition experiences and evaluating transition interventions in CAMHS and were recommended by the wider research team as potential indicators to assess participants transition experience post‐TEDYi implementation (Singh et al., 2023). Therefore, we aim to ask participants whether they think these scales are suitable and appropriate for the specific group we are targeting in terms of age and clinical presentation. Last, we will receive feedback from the participants regarding the evaluation and EBCD process. The second part of this workshop will include a dissemination event organised at King's College London (KCL) to celebrate the outcomes of the workshops and the contributions of the participants.

### 5.4 Study setting

This study will take place in four National Health Services (NHS) Trusts across England including both adolescent and adult ED specialist services. Interviews, focus groups and EBCD workshops will take place across all participating sites. Participants may instead choose to participate in the study through online interviews and focus groups on Microsoft Teams. Using multiple sites will allow further understandings and perspectives on similarities and differences in the transition process. The research assistants of the project will assist with recruitment (visiting sites to present the study objectives), virtual meetings with collaborators across the participating sites and data collection and analysis.

### 5.5 Recruitment and sampling technique

**FIGURE 1:**
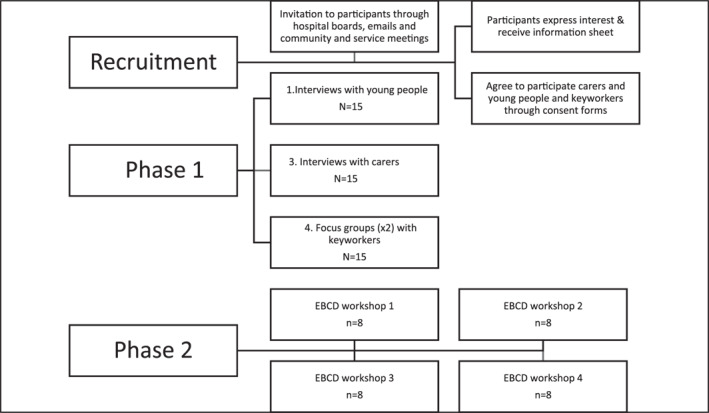
Flow diagram displaying recruitment and study tasks.

Local collaborators across four NHS Trusts and ED units will facilitate recruitment by identifying eligible young people who are close to 18 years and will be moving to adult services within the next year from the starting date of the study and/or young people who have recently transitioned to adult services or are attending an outpatient community team. The same process will be applied for eligible carers. During weekly community meetings and adverts on boards, the collaborators will invite young people to participate in the study. Young people and carers attending adult outpatient services will be contacted through post/phone by their responsible clinician. Additionally, the study will be advertised by local collaborators through leaflets/posters in ED specialist services. Young people will contact their responsible clinician if they want to participate, who will then inform the research team. In some cases, young people and their carers may be initially contacted by an invitation letter signed by their care coordinator/clinician. A follow‐up phone call/email will be made by CAMHS or Adult Mental Health Services personnel if they do not respond to the original invitation. Purposive sampling will be used to identify eligible young people, and convenience sampling for carers and mental health professionals. The sample will be proportionally stratified to represent diverse demographic characteristics if more than 15 participants are identified in each group. The sample size was decided based on information power allowing for sufficient information, diverse experiences, and new knowledge, as well as previous studies conducted with qualitative design and estimated number of eligible participants after consultation with local collaborators. Accordingly, our targeted population is expected to share sufficient information about transition experiences and will provide new knowledge for analysis. See Figure 1 for recruitment and study procedures.

### 5.6 Eligibility criteria for participants

The following inclusion criteria are for young people: (1) 16–21 years with a primary diagnosis of ED either BN or AN based on DSM‐5 criteria; AND (2) 16–21 years with EDs who are currently in TIER 3/4 ED specialist units; OR (4) 18–21 years with EDs who have moved from TIER ¾ (outpatient/inpatient care) ED specialist units to adult services and/or the community.

The following inclusion criteria are for carers: (1) carers of young people 16–21 years with EDs who are due to transition or have experienced transition to adult mental health services and the community.

Inclusion criteria for mental health professionals are as follows: (1) mental health professionals working in ED specialist adolescent units and/or adult units (nurses, psychologists, nutritionists, psychiatrists, family therapists, peer‐support workers, occupational therapists).

The following are the exclusion criteria for young people: (1) young people with EDs below 16 and over 21 years; OR (2) young people with a primary diagnosis of psychosis, and/or substance use disorder; OR (3) young people who find it difficult to communicate in English and cannot understand what is expected of them.

The following are the exclusion criteria for carers and mental health professionals: (1) lacking capacity to give informed consent due to severe morbidity (acute phase of mental illness), OR (2) not able to communicate in English

### 5.7 Data analysis

Reflexive thematic analysis (RTA) will be used to analyse the interviews and focus groups, to identify key themes regarding ED transitions. The emerging themes will then inform the EBCD workshop aims and content (Byrne, 2022). We will follow the 6‐step approach suggested by Braun and Clarke (2022) and further recent developments in RTA by Byrne (2022). Reflexive thematic analysis highlights the active role of the researcher by assigning interpretations based on personal reflections of the dataset, theoretical assumptions, and analytical skills. This study will be a collaborative process amongst researchers/co‐authors of this protocol given that multiple coders are recommended to deepen understanding of the dataset. The recordings of the interviews will be transcribed verbatim, and examined for similarities, differences, repetitions, and patterns. The predetermined codes will then be refined to produce emergent themes. The interviews video‐recorded on Microsoft Teams will be transcribed automatically and checked for accuracy. Naturally, familiarisation with the data will occur and coding of each line will take place to identify informative codes based on the participants' experience regarding their psychosocial, interpersonal, and emotional skills, barriers and facilitators of transitioning, and recommendations for the proposed intervention. A codebook will be developed based on the codes generated from the transcripts and relevant literature of transitioning from specialist ED services. The codebook will be refined with the research team and the evolution of codes will be tracked to allow returning to initial codes after future amendments. Overarching themes and subthemes will be identified based on multiple revisions of the codes and discussed in research meetings to check for interrater reliability. During the final themes reviews, the team will determine the order of the themes based on the participants narrative and priorities. Initial coding will be performed for description while second‐level “analytic” coding will follow as new themes emerge to interpret data, following RTA principles, which highlight that codes should be generated organically and should be less dependent on pre‐assumptions of the data. All thematic revisions will be documented to minimise researcher bias and a reflective diary will be kept throughout the data analysis to encourage reliability and transparency. We will aim to present participants with interview thematic analysis to receive their feedback. Latent coding will be used to account for hidden meanings of semantic context and underlying meanings of what participants shared with the research team.

A contextualist approach will be used as the main epistemological position, which lies in between constructionism and essentialism (Braun & Clarke, 2022). Analysis will account for participants' subjective experience (constructionist) of the physical reality that is transitioning to adult services and the social context of being hospitalised or attending an adolescent service (essentialist). This study will also follow an experiential orientation which will focus on the participants' experiences and meanings ascribed to their own personal narrative (Byrne, 2022). This approach will prioritise the personal reality of the participants and will lead to a meaningful co‐production process that values co‐producers’ feelings, thoughts and reflections.

### 5.8 Consent

The young person/carer/mental health professional will be required to provide consent to participate in the interviews/focus groups, for the interviews/focus groups to be recorded, and for the four follow‐up workshops. At each phase of the study, the researchers will ensure that participants have a good understanding of the risks and benefits of participating, what is expected of them, their right to withdraw during or after 4 weeks of data collection and how their data will be used to inform the proposed intervention. Ethical approval has been obtained by the UK Health Departments' Research Ethics Service (NHS RECs), London ‐ Westminster Research Ethics Committee, using the Integrated Research Application System.

## 6 ETHICAL AND REGULATORY CONSIDERATIONS

### 6.1 Assessment and management of risk

This study includes minimal risk for the young person, carer, and researcher. Four researchers from the team have lived experience and are aware of possible triggers throughout the research project. Potential risks include exposure to triggering content and experiencing distress from the study questions. Information regarding the research and requested tasks will be provided in English that is developmentally appropriate for young people 16–21 years. We will exclude young people with acute presentation of mental health disorders to mitigate high‐risk of vulnerability. The participant information sheets, and consent forms clearly indicate that participation in the study is voluntary. The forms explain the various safeguards in place, namely to liaise with a young person's clinician and/or family, if required, and the resulting impact on confidentiality. If during the study a researcher identifies or the young person discloses information that raises concern about their safety and/or the safety of others, then a detailed risk management plan is followed, which ensures that the child protection policies and procedures applicable to that service are adhered to.

### 6.2 Patient and public involvement

This protocol has been developed with the input of PPI members including three individuals with lived experience, two carers, one clinician (Lead Psychologist) and two mental health professionals from the ED team at KCL. The study documents have been reviewed by young people with lived experience 17–21 years. Young people from the Adolescent Advisory Group based at KCL and funded by NHS NIHR have reviewed the proposed study and provided feedback about the two different phases of the study. The Adolescent Mental Health Research Group is a group of young people aged 12–17 with lived experience of mental health problems who shared their views about the suitability of the TEDYi project for the targeted demographic and advised on dissemination and sharing findings. We held three virtual meetings discussing ideas and narrowing down the focus of the research. Young people with lived experience reflected on their own care pathway and identified transition to adult services and the community as a high‐risk period for relapsing due limited support in the community and access to professional help. One young person with a prior hospitalisation record highlighted that their comorbid and emerging needs were overlooked. The carers referred to lack of knowledge around the transition process which increased their anxiety on how to best support their children's needs. A Steering Group will be formed as part of the proposed study, including academics (*N* = 2), clinicians (*N* = 2), individuals with past lived experience, (*N* = 4)‐ two carers and two young people)‐, which will meet three times over the course of 12 months. The first meeting will take place prior to commencement of the study, *a*nd we will discuss current approaches to seeking consent, interview/focus groups guides, and themes informing the proposed intervention (TEDYi). The second meeting will take place 6 months after the starting date of the study and will focus on the conduct of the study and things that have worked out well with the qualitative focus groups and interviews and any concerns/issues that might have arisen such as topic sensitivity as well as adherence to protocol. During the final meeting we will review the study and reiterate the overarching themes that were generated in the EBCD phase of the project. A medical professional with clinical experience with young people with EDs will provide input on management of physical health symptoms. We will work closely with the ED Research Group and the Service User Research Enterprise at KCL to appoint one or two patient representatives. During the Steering Group meetings, we will discuss the content and structure of the proposed intervention based on identified barriers and facilitators for young people transitioning from adolescent services to the community. An appointed PPI representative will contribute to final decision making.

## 7 DISCUSSION

This protocol describes the co‐production framework of TEDYi which will be developed in partnership with young people, carers, and mental health professionals to provide a preparatory platform for adolescents transitioning to adult services. Specific initiatives for individuals with EDs co‐producing care approaches and interventions are lacking and more specifically for young people involved in ED specialist services (Lewis & Foye, 2021). To develop meaningful evidence‐based interventions, and to improve transitional processes and outcomes for young people moving from adolescent care to adult services, it is vital to involve young people with lived experience in the design and production of research projects. We aim to develop a series of empowering workshops which will support young people 16–18 years moving from adolescent ED services to adult care. TEDYi will be used as an emotional and practical tool to prepare young people and their carers 3–6 months prior to their service discharge. In future stages, we will test if TEDYi is acceptable and suitable for young people with EDs moving across services through a piloting and feasibility trial. The next phase will establish proof of concept by testing the feasibility and acceptability of TEDYi in adolescent ED services and will contribute to developing a novel model of transitional care for young people.

### 7.1 Dissemination policy

This study is registered with South London and Maudsley NHS Trust. The findings will be shared and documented in peer‐reviewed open‐access scientific journals, an internal report, and on the dedicated project website and social media platforms. All PPI members will actively participate through conference presentations, public engagement events hosted by the host organisation, and a video animation showcasing their contributions to the community and their peers. Names of participants will not appear in any written report of the study. Local collaborators will disseminate a lay person summary of the findings to all participants within a year from data collection starting date, providing an opportunity for feedback and further queries. All participatory sites will be acknowledged within any published material. The full study report will be made publicly available after the completion of data analysis. The dataset will not be published due to confidentiality reasons and protecting the rights of the participants.

## Supporting information

Supplementary Material

Supplementary Material

Supplementary Material

## Data Availability

The data that support the findings of this study are available from the corresponding author upon reasonable request.

## References

[erv3046-bib-0001] Adamson, J. , Cardi, V. , Kan, C. , Harrison, A. , Macdonald, P. , & Treasure, J. (2019). Evaluation of a novel transition support intervention in an adult eating disorders service: ECHOMANTRA. International Review of Psychiatry, 31(4), 382–390. 10.1080/09540261.2019.1573721 30916597

[erv3046-bib-0002] Arcelus, J. , Bouman, W. P. , & Morgan, J. F. (2008). Treating young people with eating disorders: Transition from child mental health to specialist adult eating disorder services. European Eating Disorders Review: The Professional Journal of the Eating Disorders Association, 16(1), 30–36. 10.1002/erv.830 17910032

[erv3046-bib-0003] Beat Eating disorders . (2020). Best practice in managing service transitions for patients affected by eating disorders. Retrieved from https://beat.contentfiles.net/media/documents/transitions‐best‐practice‐final.pdf

[erv3046-bib-0004] Berends, T. , Boonstra, N. , & Van Elburg, A. (2018). Relapse in anorexia nervosa: A systematic review and meta‐analysis. Current Opinion in Psychiatry, 31(6), 445–455doi. 10.1097/YCO.0000000000000453 30113325

[erv3046-bib-0005] Braun, V. , & Clarke, V. (2022). Conceptual and design thinking for thematic analysis. Qualitative Psychology, 9(1), 3–26. 10.1037/qup0000196

[erv3046-bib-0006] Bruch, H. (2001). The golden cage: The enigma of anorexia nervosa. Harvard University Press.

[erv3046-bib-0007] Bryan, D. C. , Macdonald, P. , Cardi, V. , Rowlands, K. , Ambwani, S. , Arcelus, J. , Bonin, E. M. , Landau, S. , Schmidt, U. , & Treasure, J. (2022). Transitions from intensive eating disorder treatment settings: Qualitative investigation of the experiences and needs of adults with anorexia nervosa and their carers. BJPsych Open, 8(4), e137. 10.1192/bjo.2022.535 35856250 PMC9347315

[erv3046-bib-0008] Byrne, D. (2022). A worked example of Braun and Clarke's approach to reflexive thematic analysis. Quality and Quantity, 56(3), 1391–1412. 10.1007/s11135-021-01182-y

[erv3046-bib-0009] Cardi, V. , Ambwani, S. , Robinson, E. , Albano, G. , MacDonald, P. , Aya, V. , Rowlands, K. , Todd, G. , Schmidt, U. , Landau, S. , Arcelus, J. , Beecham, J. , & Treasure, J. (2017). Transition care in anorexia nervosa through guidance online from peer and carer expertise (TRIANGLE): Study protocol for a randomised controlled trial. European Eating Disorders Review, 25(6), 512–523. 10.1002/erv.2542 28944595

[erv3046-bib-0010] Eddy, K. T. , Tabri, N. , Thomas, J. J. , Murray, H. B. , Keshaviah, A. , Hastings, E. , Krishna, M. , Herzog, D. B. , Franko, D. L. , & Keel, P. K. (2017). Recovery from anorexia nervosa and bulimia nervosa at 22‐year follow‐up. Journal of Clinical Psychiatry, 78(2), 17085–17189. 10.4088/JCP.15m10393 PMC7883487 28002660

[erv3046-bib-0011] Eisler, I. , Simic, M. , Fonagy, P. , & Bryant‐Waugh, R. (2022). Implementing service transformation for children and adolescents with eating disorders across England: The theory, politics, and pragmatics of large‐scale service reform. Journal of Eating Disorders, 10(1), 1–15. 10.1186/s40337-022-00665-z 36217209 PMC9549853

[erv3046-bib-0012] Giel, K. E. , Behrens, S. C. , Schag, K. , Martus, P. , Herpertz, S. , Hofmann, T. , Skoda, E. M. , Voderholzer, U. , von Wietersheim, J. , Wild, B. , Zeeck, A. , Schmidt, U. , Zipfel, S. , & Junne, F. (2021). Efficacy of post‐inpatient aftercare treatments for anorexia nervosa: A systematic review of randomized controlled trials. Journal of eating disorders, 9, 1–13. 10.1186/s40337-021-00487-5 34654471 PMC8518230

[erv3046-bib-0013] Hayes, H. , Buckland, S. , & Tarpey, M. (2012). Involve: Briefing notes for researchers: Public involvement in NHS, public health and social care research. NHS‐National Institute for Health Research.

[erv3046-bib-0014] Hibbs, R. , Rhind, C. , Salerno, L. , Lo Coco, G. , Goddard, E. , Schmidt, U. , Micali, N. , Gowers, S. , Beecham, J. , Macdonald, P. , Todd, G. , Campbell, I. , & Treasure, J. (2015). Development and validation of a scale to measure caregiver skills in eating disorders. International Journal of Eating Disorders, 48(3), 290–297. 10.1002/eat.22362 25351932

[erv3046-bib-0015] Lewis, H. K. , & Foye, U. (2021). From prevention to peer support: A systematic review exploring the involvement of lived‐experience in eating disorder interventions. Mental Health Review Journal, 27(1), 1–17. 10.1108/MHRJ-04-2021-0033

[erv3046-bib-0016] Lovibond, P. F. , & Lovibond, S. H. (1995). The structure of negative emotional states: Comparison of the depression anxiety stress scales (DASS) with the beck depression and anxiety inventories. Behaviour Research and Therapy, 33(3), 335–343. 10.1016/0005-7967(94)00075-U 7726811

[erv3046-bib-0017] Madden, M. , Morris, S. , Ogden, M. , Lewis, D. , Stewart, D. , & McCambridge, J. (2020). Producing co‐production: Reflections on the development of a complex intervention. Health Expectations, 23(3), 659–669. 10.1111/hex.13046 32233053 PMC7321726

[erv3046-bib-0018] National Health Services (NHS) England . (2015). Access and waiting time standard for children and young people with an eating disorder. Retrieved from https://www.england.nhs.uk/wp‐content/uploads/2015/07/cyp‐eating‐disorders‐access‐waiting‐time‐standard‐comm‐guid.pdf

[erv3046-bib-0019] NHS England (2019). The NHS long term plan. NHS. Retrieved from https://www.long‐termplan.nhs.uk/wp‐content/uploads/2019/08/nhs‐long‐term‐plan‐%20version‐1.2.pdf

[erv3046-bib-0020] Norton, M. (2019). Implementing co‐production in traditional statutory mental health services. Mental Health Practice. 10.7748/mhp.2019.e1304

[erv3046-bib-0021] Patel, K. , Tchanturia, K. , & Harrison, A. (2016). An exploration of social functioning in young people with eating disorders: A qualitative study. PLoS One, 11(7), e0159910. 10.1371/journal.pone.0159910 27458808 PMC4961427

[erv3046-bib-0022] Rienecke, R. D. (2018). Expressed emotion and eating disorders: An updated review. Current Psychiatry Reviews, 14(2), 84–98. 10.2174/1573400514666180808115637

[erv3046-bib-0023] Royal College of Psychiatrists (2022). Delivering better outcomes for children and young adults –new service models and better transitions across mental health. Retrieved from https://www.rcpsych.ac.uk/docs/default‐source/improving‐care/better‐mh‐policy/position‐statements/ps03_22.pdf?sfvrsn=23c278e0_8

[erv3046-bib-0024] Sepulveda, A. R. , Whitney, J. , Hankins, M. , & Treasure, J. (2008). Development and validation of an eating disorders symptom impact scale (EDSIS) for carers of people with eating disorders. Health and Quality of Life Outcomes, 6, 1–9. 10.1186/1477-7525-6-28 18426597 PMC2365933

[erv3046-bib-0025] Singh, S. P. , Tuomainen, H. , Bouliotis, G. , Canaway, A. , De Girolamo, G. , Dieleman, G. C. , Franić, T. , Madan, J. , Maras, A. , McNicholas, F. , Paul, M. , Purper‐Ouakil, D. , Santosh, P. , Schulze, U. M. E. , Street, C. , Tremmery, S. , Verhulst, F. C. , Wells, P. , Wolke, D. , Warwick, J. , & MILESTONE, Consortium (2023). Effect of managed transition on mental health outcomes for young people at the child‐adult mental health service boundary: A randomised clinical trial. Psychological Medicine, 53(6), 2193–2204. 10.1017/S0033291721003901 37310306 PMC10123823

[erv3046-bib-0026] Treasure, J. , Crane, A. , McKnight, R. , Buchanan, E. , & Wolfe, M. (2011). First do no harm: Iatrogenic maintaining factors in anorexia nervosa. European Eating Disorders Review, 19(4), 296–302. 10.1002/erv.1056 21714039

[erv3046-bib-0027] Treasure, J. , & Schmidt, U. (2013). The cognitive‐interpersonal maintenance model of anorexia nervosa revisited: A summary of the evidence for cognitive, socio‐emotional and interpersonal predisposing and perpetuating factors. Journal of eating disorders, 1(1), 1–10. 10.1186/2050-2974-1-13 24999394 PMC4081714

[erv3046-bib-0028] Tsianakas, V. , Maben, J. , Wiseman, T. , Robert, G. , & Richardson, A. (2010). Using Experience‐based co‐design (EBCD) to improve breast and lung cancer services. The European Journal of Public Health, 20, 63. 10.1007/s00520-012-1470-3 PMC3461206 22544223

[erv3046-bib-0029] Wales, J. , Brewin, N. , Susi, K. , Eivors, A. , Whight, D. , & Leatherland, R. (2021). Experience of transition between a child and adolescent service and adult service for the treatment of eating disorders. Mental Health Review Journal, 26(2), 128–142. 10.1108/MHRJ-01-2020-0005

[erv3046-bib-0030] Walker, S. , & Lloyd, C. (2011). Barriers and attitudes health professionals working in eating disorders experience. International Journal of Therapy and Rehabilitation, 18(7), 383–390. 10.12968/ijtr.2011.18.7.383

[erv3046-bib-0031] Webb, H. , & Schmidt, U. (2021). Facilitators and barriers to supporting young people with eating disorders during their transition to, and time at, university: An exploration of clinicians' perspectives. European Eating Disorders Review, 29(3), 443–457. 10.1002/erv.2795 33044033

[erv3046-bib-0032] Winston, A. P. , Child, S. , Jackson, J. , & Paul, M. (2023). Management of transitions to adult services for young people with eating disorders: Survey of current practice in England. BJPsych Bulletin, 47(1), 17–22. 10.1192/bjb.2021.109 34994343 PMC10028553

[erv3046-bib-0033] Wood, D. L. , Sawicki, G. S. , Miller, M. D. , Smotherman, C. , Lukens‐Bull, K. , Livingood, W. C. , Ferris, M. , & Kraemer, D. F. (2014). The transition readiness assessment Questionnaire (TRAQ): Its factor structure, reliability, and validity. Academic pediatrics, 14(4), 415–422. 10.1016/j.acap.2014.03.008 24976354

[erv3046-bib-0034] Zaremba, N. , Robert, G. , Allan, J. , Harrison, A. , Brown, J. , Konstantara, E. , Rosenthal, M. , Pillay, D. , Beckwith, A. , Treasure, J. , Hopkins, D. , Ismail, K. , & Stadler, M. (2022). Developing a novel intervention for type 1 diabetes and disordered eating using a participatory action design process: Safe management of people with Type 1 diabetes and EAting Disorders studY (STEADY). Diabetic Medicine, 39(4), e14749. 10.1111/dme.14749 34821402

[erv3046-bib-0035] Zigmond, A. S. , Snaith, R. P. , & Kitamura, T. (1983). The hospital anxiety and depression scale (HADS). Acta Psychiatrica Scandinavica, 67(6), 361–370. 10.1111/j.1600-0447.1983.tb09716.x 6880820

