# Supplementary material for: Co‐producing an inclusive‐care model for young people transitioning from adolescent eating disorder services to adult care: A qualitative study protocol for Transition for Eating Disorder Youth intervention
Source: Eur Eat Disord Rev. 2023 Nov 7;34(1):5–16. doi: 10.1002/erv.3046 (PMC12694689; doi:10.1002/erv.3046)
Supplement: Supplementary file 1 — Supplementary Material [file ERV-34-5-s001.docx]

***
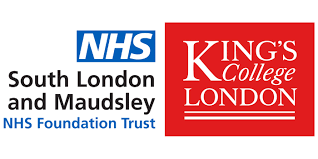
***

**Study Title**: Co-producing an inclusive-care model for young people transitioning from adolescent eating disorder services to adult care: Transition for Eating Disorder Youth intervention (TEDYi)

**Topic List: Focus group schedules for mental health professionals**

***Introduction***

Thank you for agreeing to be interviewed today. My name is_____________
and I am a researcher based at King’s College London. We are doing a study looking at what happens when a person who is attending an eating disorder specialist service, has their care transferred to an adult mental health service.

As a mental health professional involved in the transitional care of young people who are about to move or have moved from one service to another, we would like to talk to you today about your experiences with adolescent and adult eating disorder services. This will help us to develop ideas on how to improve services, especially for people and their carers who may have to move from one service to another in the future.

***Schedule for mental health professionals***

- I would like to remind you that anything you tell me will remain confidential. The only situation where this would not apply is if you told me something that made me concerned that there was a risk of serious harm to either yourself or to another person.
- All the information collected from today will be stored on a computer with each person identified only by a number code. Only the researchers involved in the study will be able to view the information and when this information is used in future reports and publications no one will be able to recognise you from the information.
- Are you willing for me to video-record our conversation so that I don’t have to write while we are talking? As you have consented, your information might be used and shared in the second phase of this study.
- To make the research most useful, I need to know both positive and negative things so please don’t hesitate to tell me if you have any problems to report. The comments from everyone who is interviewed are combined anonymously when the results are reported so no one can be identified.

• ***Consent form.***

**Section 1: Achieving Successful Transition from CAMHS to Adult Mental Health Services**

- What is the current process for ensuring successful transition from CAMHS to Adult Mental Health Services?
  - What is your role in the process? Beginning and ending of role boundary?
  - What is the cut-off age or criteria for end of CAMHS and starting age or criteria for take up of adult mental health services in the Trust? Is there discharge planning?
- Could you tell me what your service does once it is decided that a young person needs to transfer to another service and, specifically to an adult service?
  - *(Prompts: What is the process about making the referral?
    on what criteria do you decide what service they are transferred to? Why?*
  - *What is your ideal of a good transfer of care?*
  - *Which aspects of transitional care do young people receive?*
  - *Any difficulties in accessing specific adult service?*
  - *What do CAMHS do to help young people with transition?*
- What services are available to service users/carers during transition? Range of services (including dual diagnosis). Geographical boundaries? Links to voluntary organisations?
- How do CAMHS transitions differ from adult transitions?
- How do the range and availability of services meet user/carer needs?
- Availability of policies and guidelines to staff to inform the process of transition?
  - Team meetings between CAMHS and Adult Services?
  - Collaborative decision making?
  - Communication of decisions to support transition?
- Arrangements and mechanisms for following up with service users/carers or teams where transition has not occurred or there are problems? How effective are these?
- Resources to support transition? Human resources in teams? Shortages? Use of temporary staff? Access to information and computer equipment?
- What are the greatest challenges to achieving successful transition in the way services are currently organised?

**Section 2: Preparing and engaging service users for and in transitional arrangements**

- How are individual users/carers prepared for and engaged in transitional arrangements?
- How are they involved in decisions about meeting their needs? What about family’s role?
- Are there any areas where this might be improved?
- Examples where transition has worked well and why.
- Examples where transition has worked less well and why.

**Section 3: Barriers and facilitators to achieving successful transition?**

What are the barriers to achieving successful transition?

• Most common three?

• How to reduce/overcome these?

• Why do you think these barriers exist?

• What would help you to overcome them?

What are the facilitators (success factors) in achieving successful transition?

• Most common?

• How to promote/sustain these?

• Availability to you?

**Section 4: Inter-agency Working**

How do you manage/promote interagency working during transition?

• Priorities in achieving this?

• Approaches to decision making?

• How does this impact on achieving transition?

• Please give examples based on your experience.

- In your opinion, has the process of changing from CAMHS to adult services had an effect on young people?
  - *(Prompts:
    Independence from parents,*
  - *engagement with services,*
  - *understanding of problems and effects on severity of mental health problems-**Better?,* *Worse?,*
  - *Any new problems?)*
- Is there anything else you would like to mention that we haven’t talked about yet?
