# Supplementary material for: Co‐producing an inclusive‐care model for young people transitioning from adolescent eating disorder services to adult care: A qualitative study protocol for Transition for Eating Disorder Youth intervention
Source: Eur Eat Disord Rev. 2023 Nov 7;34(1):5–16. doi: 10.1002/erv.3046 (PMC12694689; doi:10.1002/erv.3046)
Supplement: Supplementary file 2 — Supplementary Material [file ERV-34-5-s002.docx]

***
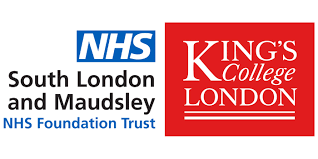
***

**Study Title**: Co-producing an inclusive-care model for young people transitioning from adolescent eating disorder services to adult care: Transition for Eating Disorder Youth intervention (TEDYi)

**Topic List: Interview Schedules for Carers**

***Introduction***

Thank you for agreeing to be interviewed today. My name is_____________
and I am a researcher based at King’s College London. We are doing a study looking at what happens when a person who is attending an eating disorder service, has their care transferred to an adult eating disorder service.

As your child is about to move or has moved from one service to another, we would like to talk to you today about your experiences with adolescent (and adult if applicable) eating disorder services. This will help us to develop ideas on how to improve services, especially for people who may have to move from one service to another in the future and their carers.

***Schedule for parents***

- I would like to remind you that everything you tell me will remain confidential. The only situation where this would not apply is if you told me something that made me concerned that there was a risk of serious harm to either yourself or to another person.
- All the information collected from today will be stored on a computer with each person identified only by a number code. Only the researchers involved in the study will be able to view the information and when this information is used in future reports and publications no one will be able to recognise you from the information.
- Are you willing for me to video-record our conversation so that I don’t have to write while we are talking? As you have consented, your information might be used and shared in the second phase of this study. However, we will not include any quotes or information in the filmed version that you would not like to share.
- To make the research most useful, I need to know both positive and negative things so please don’t hesitate to tell me if you have any problems to report. The comments from everyone who is interviewed are combined anonymously when the results are reported so no one can be identified.
- Please let me know if you need to take a break during the interview.
- You don’t need to answer anything you do not feel comfortable with.

• ***Consent form.***

**1. Child and Adolescent services–entry, illness course and overall experience**

- Could you tell me the story about how your child first came to Child and Adolescent Mental Health Services?
  - *(Prompts: What was the problem?*
  - *Who asked him/her to be seen there and why?*
  - *How old was s/he?)*
- What is their diagnosis? /Did they have any other diagnosis?
- Could you tell me about your and your child’s experiences of using Child and Adolescent Mental Health Services?
  - *(Prompts: What happened at CAMHS?*
  - *Can you think of anything particularly helpful? Was there anything unhelpful?*
  - *Is there anything you would change?*
  - *How involved are you/were you?*

**2. Transition Planning**

- How did you realise that your child would have to move from Child and Adolescent Mental Health Services to the Adult services?
- What are/were your thoughts about your child moving to adult care?
- What is your role as a carer in the process?
  - How have you been/or not been involved?
- What kind of support have you or are you receiving about your child’s prospective move to adult services?
- Was there anything that helped or was unhelpful in preparing you for this move?
- Thinking back, is there anything that would have been more helpful in preparing you and your child for the move, or anything that you would change?
  - What can be done differently to make the transition easier?

**3. Transition issues**

- What do you think were the main reasons why your child was referred to adult services?
- Was the reason something that makes sense to you?
- Thinking about you and your family, what would be good reasons for your child to move from the Child and Adolescent Mental Health Services to the Adult services?
- How far in advance were you told of the potential transition? Was it an open conversation you were heavily involved in?

*(Prompt: If not, why do you think this was? Was it harmful in any way?*

**4. Adult services – entry, engagement and defaulting, and overall experience (if applicable to those who have moved to adult care)**

- Have you or your child been to the adult service you were referred to?
  - *(Prompts: If so ‘in what ways?’ If no, ‘why not?’)*
- What do/did you know about transition planning/management/preparation?
- What has it been like going there?
- Were you involved in decision-making? Did you have a choice with regards the geographical location? (e.g., accessibility, travel costs)

**5. Comparison of Adult to Child and Adolescent services (if applicable)**

- What have you found to be the main differences in adult services as compared to the child and adolescent services?
- Are there any ways in which it has been better/easier/more helpful going to the adult service than CAMHS?
- Are there any ways in which CAMHS was better/easier/more helpful than going to than the adult service?
- Has the transition to Adult services altered your relationship with your child in any way? *(Better/worse, why/why not?)*

**6. Potential impact of transition**

- In your opinion, has the process of changing from CAMHS to AMHS had any effect on you or your child?
  - *(Prompts:
    Independence from parent*
  - *Engagement with services*
  - *Understanding of problems*
  - *Effects on severity of mental health problems-**Better?,* *Worse?, Any new problems?*
- What are the challenges you think you might come across after CAMHS discharge?
- What are your and your child’s plans after leaving CAMHS?
- Is there anything else you would like to say about the transition from CAMHS to adult services that we haven’t talked about yet?
