# Supplementary material for: Co‐producing an inclusive‐care model for young people transitioning from adolescent eating disorder services to adult care: A qualitative study protocol for Transition for Eating Disorder Youth intervention
Source: Eur Eat Disord Rev. 2023 Nov 7;34(1):5–16. doi: 10.1002/erv.3046 (PMC12694689; doi:10.1002/erv.3046)
Supplement: Supplementary file 3 — Supplementary Material [file ERV-34-5-s003.docx]

***
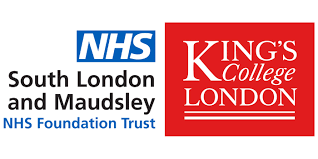
***

**Study Title**: Co-producing an inclusive-care model for young people transitioning from adolescent eating disorder services to adult care: Transition for Eating Disorder Youth intervention (TEDYi)

**Topic List: Interview Schedules for Young People**

***Introduction***

Thank you for agreeing to be interviewed today. My name is_____________
and I am a researcher based at King’s College London. We are doing a study looking at what happens when a person who is attending an eating disorder service, has their care transferred to adult services. As you are about to move or have moved from one service to another, we would like to talk to you today about your experiences with adolescent (and adult if applicable) eating disorder services. This will help us to develop ideas on how to improve services, especially for people who may have to move from one service to another in the future and their carers.

***Schedule for young people***

- I would like to remind you that anything you tell me will remain confidential. The only situation where this would not apply is if you told me something that made me concerned that there was a risk of serious harm to either yourself or to another person.
- All the information collected from today will be stored on a computer with each person identified only by a number code. Only the researchers involved in the study will be able to view the information and when this information is used in future reports and publications no one will be able to recognise you from the information.
- Are you willing for me to video-record our conversation so that I don’t have to write while we are talking? As you have consented, your information might be used and shared in the second phase of this study. However, we will not include any quotes or information in the filmed version that you would not like to share.
- To make the research most useful, I need to know both positive and negative things so please don’t hesitate to tell me if you have any problems to report.
- The comments from everyone who is interviewed are combined anonymously when the results are reported so no one can be identified.
- Please let me know if you need to take a break during the interview.
- You don’t need to answer anything you do not feel comfortable with.

• ***Consent form.***

**1. Child and Adolescent services–entry, illness course and overall experience**

- Could you tell me the story about how you first came to Child and Adolescent Mental Health Services?
  *(Prompts: Who asked you to be seen there and why?*
  *How old were you?)*
- Could you tell me about your experiences of using Child and Adolescent Mental Health Services for Eating Disorders?
  - *(Prompts: What happened at CAMHS?*
  - *Was there anything helpful? Was there anything unhelpful?*
  - *Is there anything you would change?)*
- What is your diagnosis?
- Did you receive any other diagnosis apart from ED? /Did you receive treatment for that?
- What treatment did you receive when attending CAMHS? (helpful/unhelpful?)
- How much was your view taken into account throughout the process? (*Did you feel able to put your view forward, if so why/why not?)*

**2. Transition Planning**

- How did you realise that you would have to move from Child and Adolescent Mental Health Services to the Adult service?
- What are your thoughts about moving to adult care?
- What kind of support have you or are you receiving about your prospective move to adult services?
- Was there anything that helped or was unhelpful in preparing you for this move?
- What skills would have been/will be particularly useful for you to learn during the transition?
- Thinking back, is there anything that would have been more helpful in preparing you for the move, or anything that you would change?
  - What could have been done differently to make your move easier?
  - Is there anything that was particularly helpful or encouraging prior to the transition?
  - Was there anything that really impaired/will impair your transition?

**3. Transition issues**

- What do you think were the main reasons why you were referred to adult services?
- Was the reason something that makes sense to you? How did you feel about this?
- How did you initially feel when your transition to adult services was discussed?
  - Did you have an insight as to what this would involve?
  - Did you have any pre-existing positive or negative thoughts about transitioning?
    - If yes, from where?
- Have you had experienced any previous service/school/family transitions before? (What was your experience? Reflections?)
- Thinking about you and your family, what would be good reasons for you to move from the Child and Adolescent Mental Health Services to the Adult services?

**4. Adult services – entry, engagement and defaulting, and overall experience (if applicable to those who have moved to adult care)**

- Have you been to the adult service you were referred to?
- What treatment are you receiving/will you be receiving within adult services (if you know)?

*(Prompts: If so ‘in what ways?’ If no, ‘why not?’)*

- What has it been like going there?
- What was it that encouraged you to engage with the referred adult services?
- What have you found to be the main differences in adult services as compared to the child and adolescent services?

**5. Comparison of Adult to Child and Adolescent services**

- Are there any ways in which it has been better/easier/more helpful going to the adult service than CAMHS?
- Are there any ways in which CAMHS was better/easier/more helpful than going to than the adult service?
- Did the communication differ between Adult services and Child and Adolescent services, if so, to what extent? *(Prompt: Significantly more/less information shared? Types of communication styles?)*

**6. Potential impact of transition**

- In your opinion, has the process of changing from CAMHS to adult care had any effect on you?
- What are the challenges you think you might come across after CAMHS discharge?
  - *(Prompts: Independence from parent*
  - *Engagement with services*
  - *Understanding of problems*
  - *Effects on severity of mental health problems-**Better?,* *Worse?, Any new problems? If so, why do you think that is?*
- What are you doing now? (college/working/hobbies, etc) OR What are your plans once you leave CAMHS?
- Is there anything else you would like to say about the transition from CAMHS to adult services that we haven’t discussed yet?

**7. Family and Carer involvement**

- How were your family/carers involved in your transition?
  - (Prompts: was their involvement positive or negative for you? In what ways? Anything you would have wanted to be different?)
- Ideally, which person in your life would you prefer to be involved in your transition and why?
- How has your relationship with your carer been during your transition?
  - (Prompts: has anything changed, positively or negatively)

**Only for the ones who moved to adult services already.**

- Thinking about the ways in which your family/carers were involved in your treatment at CAMHS, how is their involvement different or the same in the adult services?
  - What implications has this had, if any?
  - Would you have wanted anything regarding their involvement to be different?
- Has your relationship with your carer changed since you have been in the adult services?
  - If yes how?
  - If yes, has it impacted you in any way? Has it impacted your treatment progress? If yes how?
